# Breastfeeding and its impact on daily life in women with type 1 diabetes during the first six months after childbirth: a prospective cohort study

**DOI:** 10.1186/1746-4358-7-20

**Published:** 2012-12-21

**Authors:** Marie Berg, Lena-Karin Erlandsson, Carina Sparud-Lundin

**Affiliations:** 1 Institute of Health and Care Sciences, Sahlgrenska Academy, University of Gothenburg, Gothenburg, Sweden; 2 Department of Health Sciences, Faculty of Medicine, Lund University, Lund, Sweden

## Abstract

**Background:**

For mothers with diabetes, breastfeeding is a great challenge due to their struggle with potentially unstable blood glucose levels. This paper explores breastfeeding attitudes and impact of breastfeeding on the daily life of mothers with type 1 diabetes compared with non-diabetic mothers.

**Methods:**

We performed a prospective cohort study of 108 mothers with type 1 diabetes and a reference group of 104 mothers in the west of Sweden. Data were collected through medical records and structured telephone interviews at 2 and 6 months after childbirth.

**Results:**

Women in both the diabetes group and the reference group had high levels of confidence (84% and 93% respectively) in their breastfeeding capacity before childbirth, and 90% assessed breastfeeding as a positive and an important experience during the six months of follow-up. About 80% assessed breastfeeding as influencing daily life ‘very much’ or ‘quite a lot’ at 2 months as did 60% at 6 months, with no difference between the groups. In mothers with diabetes, the impact of breastfeeding on the priority of other duties decreased over time, as did feelings of time pressure and negative effects on patterns of sleep. Compared to the reference group, mothers with diabetes at 6 months remained more affected by disruptions in daily life and they felt more worried about their health both at 2 and 6 months after childbirth. For the reference group mothers’ sensitivity to unexpected disruptions in daily routines decreased between 2 and 6 months after childbirth, and they expressed a greater need to organize their time than mothers with diabetes.

**Conclusion:**

Mothers with diabetes type 1 express more worry for own health and are more sensitive to distruptions. To balance their everyday life and to reduce the risk of stress and illhealth they are therefor, compared to other mothers, likely to need additional professional and peer support.

## Background

It is recommended that mothers should exclusively breastfeed their infants for the first 6 months because of the many benefits of breastfeeding [1]. For mothers, breastfeeding is a deeply personal and embodied experience which requires maternal commitment, adaption and support [2]. For some mothers, breastfeeding leads to feelings of connectedness including harmony, oneness, and completeness, while for others it can lead to disconnectedness and dissatisfaction [3] and be seen as a moral imperative [4]. Breastfeeding influences self-esteem [5] but for many women it is connected with shame and guilt related to a feeling of failure to live up to ideals of good motherhood [6]. Mothers who exclusively breastfeed report higher levels of self-efficacy than mothers who exclusively formula feed their infants [7]. Several studies report high maternal breastfeeding self-efficacy being associated with prolonged breastfeeding [8], and women’s intention to breastfeed to be a strong predictor for breastfeeding initiation and duration [9].

For mothers with diabetes, breastfeeding may be more challenging than for mothers without diabetes. The initiation and establishment is often affected by birth related complications [10,11]; their newborns often show a premature sucking pattern [12] and often need early feeding because of a high risk of hypoglycaemia [13,14]. Early initiation of breastfeeding is shown to be a strong predictive factor for long-term breastfeeding of infants whose mothers have type 1 diabetes [10,11].

Mothers with type 1 diabetes have a different point of entry into the breastfeeding situation than non-diabetic mothers. Their situation includes a struggle in daily life with breastfeeding, albeit with a strong motivation to succeed, simultaneously filled with uncertainty and unpredictability related to their own unstable glycemic control [15,16]. This instability results from a decreased need for insulin related to increased glucose consumption during the first few weeks of breastfeeding [17,18]. Mothers with diabetes experience daily life as unpredictable and related to problems with recognising their signs and symptoms of bodily reactions related to diabetes during breastfeeding [15,16].

Activities in daily life have been described as forming a pattern [19]. Some activities dominate in terms of time and awareness, such as working or watching a movie, while others are performed with less attention, such as driving to work or having a cup of coffee. Daily life also includes a varied number of unexpected activities which interrupt and change the rhythm in the pattern of daily activities. Whilst most adults organize their daily life around stable weekly rhythms of activities, they are to some extent open to changes of plans and routines. Thus, people can generally manage with having a daily life and patterns of daily activities that are only partly predictable [19]. However, having diabetes implies a tension between balancing self-management of diabetes with engagement in valued activities, and predictability and stable routines are beneficial for self-management of the disease [20]. For mothers with diabetes, greater effort and flexibility is required when they become a mother in order to balance glycemic control because breastfeeding impacts on the patterns of daily activities and often requires establishing new daily patterns of activities [15,21]. With the aim of identifying needs to develop supportive activities, this paper explores breastfeeding attitudes and the impact of breastfeeding on the daily life of mothers with type 1 diabetes compared with non-diabetic mothers.

## Methods

As part of a research project investigating breastfeeding in mothers with type 1 diabetes (MODIAB-breastfeeding) a prospective cohort study comparing diabetes group with a reference group was performed. Findings have been reported in two publications [11,22].

### Participants and setting

Mothers with type 1 diabetes who had given birth during 2007–2009 at four hospitals in the western region of Sweden were invited to participate in the study by a clinical midwife assisting the research project. The women were invited during their stay at the maternity ward. For every included mother with type 1 diabetes, the next mother giving birth at the same hospital was invited as a reference if she did not have any form of diabetes or other long-term illness, and if she fulfilled the matching criteria: gestational week and same parity (primipara or multipara). Due to their short stay at the maternity ward, these reference mothers were contacted by the clinical research midwife by telephone within two weeks after childbirth. Exclusion criteria for both groups were inability to understand and speak Swedish. Verbal and written information about the study was given and informed consent was obtained from each participant. The study was approved by the Regional Ethical Review Board (Protocol 351–07).

In Sweden, a team of midwives, obstetricians and pediatricians manages the care of mother and child during the first week after childbirth. Thereafter the primary health care organization is responsible for the health care of children aged 0–6 years. This child health care service is free of charge and almost all parents visit the pediatric/district nurse/physician regularly during this time, with high frequency in the first few months and with longer intervals thereafter.

### Data collection

Data on mode of delivery, gestational week and parity were collected from medical records. In addition, data were collected from telephone interviews with the mothers at 2 and 6 months after childbirth. The interviews followed a structured questionnaire developed by the research group and based on earlier studies [15,23,24]. The questionnaire had been tested for face validity including comprehensibility and meaningfulness in a pilot group of 10 women with type 1 diabetes and 10 matched controls. Included were questions on socio-demographic factors, confidence about breastfeeding capacity (‘very high’/‘high’/‘low’), overall experience of breastfeeding (positive/negative), attitude to the importance of breastfeeding (‘very important’/‘important’/‘quite important’/‘unimportant’); occurrence of complications such as milk supply, occurrence of blocked ducts, cracked nipples or painful breastfeeding (yes/no). Agreement with a series of statements on how breastfeeding had impacted on daily structure (yes/no) between 0–2 months and between 2–6 months were also included, as previous qualitative studies had identified that mothers with diabetes struggle to get structure in daily life with simultaneous demands of diabetes management and breastfeeding [15]. An example of such statement is “breastfeeding has decreased the priority of other duties” (see all statements in Table 1).

**Table 1:** Comparison of breastfeeding impact on daily structure in mothers with and without type 1 diabetes 0–2 and 2–6 months postpartum

	**Mothers in Diabetes Group (DG)**		**Mothers in Reference Group (RG)**		**DG versus RG**	
Agreement with statements on whether breastfeeding had impacted on daily structure (yes/no*)	0-2 months** postpartum*n* = 105	2-6months** postpartum*n* = 97	0-2 months** postpartum*n* = 101	2-6months** postpartum*n* = 94	**0-2 months postpartum*p* value**^**2**^	**2-6 months postpartum*p* value**^**2**^
It has decreased the priority of *n* (%) other duties	87 (71.4)	52 (53.6)	71 (70.3)	64 (68.5)	0.879	0.076
I sleep better than before *n* (%)	7 (6.7)	5 (8.2)	9 (8.9)	6 (6.8)	0.609	0.783
I sleep worse than before *n* (%)	81 (77.1)	62 (63.9)	83 (82.2)	70 (74.2)	0.392	0.165
I need to organize my time more than before *n* (%)	87 (82.9)	71 (73.2)	94 (93.1)	82 (87.6)	0.032	0.051
I have become more sensitive to unexpected disruptions in my daily routine *n* (%)	57 (54.3)	49 (50.5)	48 (47.5)	31 (33.0)	0.403	0.041
I feel more worried about my health *n* (%)	36 (34.3)	31 (32.0)	10 (10.9)	14 (14.8)	<0.001	0.012
I feel less worried about my health *n* (%)	27 (25.7)	19 (19.6)	28 (27.7)	24 (25.0)	0.755	0.598
I feel time pressure in my daily life *n* (%)	78 (74.3)	58 (59.8)	62 (61.4)	55 (58.4)	0.053	1.000
I feel more calm and harmonious than before *n* (%)	46 (43.8)	40 (41.7)	44 (44.0)	42 (44.3)	1.000	1.000

^1^ Wilcoxon Signed Rank Test ^2^ Fisher’s exact test ^*^only ‘yes’ answers are reported ** experiences concerned breastfeeding impact during the period.

In this project, exclusive breastfeeding was defined as no supplementation of formula milk, and partial breastfeeding as a combination of breastfeeding and formula feeding. To distinguish between the occurrence of exclusive and partial breastfeeding, the women were asked at time for telephone interview (at 2 and 6 months postpartum), how many weeks (or months) after breastfeeding initiation they had breastfed exclusively and partially respectively (data not reported here).

### Data analysis

Statistical power calculations were calculated on the variables, breastfeeding rates [11] and psychological well-being [22] and not the variables central in this study. Analyses of study variables were conducted using the software SPSS 18.0 (Chicago, IL). Tests were two-tailed and conducted at 5% significance level. Mean (standard deviation/SD), median, and range (min-max) was used for descriptive data; n (%) was used for categorical and dichotomous variables. Pearson’s Chi-Square test and Fisher’s Exact test were used for dichotomous variables to analyse differences between the diabetic group (DG) and the reference group (RG). Wilcoxon Signed Rank test was used to evaluate differences over time within the groups, e.g. between measurements at 2 and 6 months after childbirth.

## Results

### Study group characteristics

Included at 2 months were 108 mothers in the diabetic group and 104 in the reference group: at 6 months these were 108 and 99 respectively. The number of potential participants in the diabetic group was 128. Those who declined to participate or missed inclusion (n = 20) did not differ in any of the following aspects: age, gestational week, mode of delivery, and birth weight. It was not possible to conduct attrition analysis on the reference group due to logistical obstacles. Study group characteristics have been presented elsewhere [11]. In brief, mean age did not differ between the two groups (31.2 vs. 31.1, p = 0.88) and neither did marital status: 97.2% vs. 98.1% were co-habiting (p = 0.37). Moreover, educational level did not differ between the groups (p = 0.93). The majority had completed university degrees (diabetes group 56.1% and reference group 58.3%). Mean gestational week at childbirth in the total group (diabetes and reference group) was 37.9 (SD 1.8) and 53% were primiparous with no differences between the groups in line with the matching criteria. Vaginal delivery was less common among mothers with diabetes than in the reference group (43.1 vs. 74.3%) [11].

The frequency of breastfeeding at discharge from hospital was 55% in the diabetic group compared to 76.2% in the reference group (p < 0.01). Breastfeeding rates at 2 and 6 months after childbirth are presented in Table 2, and originally in Sparud-Lundin et al. [11].

**Table 2:** Comparison of breastfeeding rates, complications and experience in mothers with type 1 diabetes (DG) and the reference group (RG)

**Variables**	**Diabetes group *0*–*2 months n = 108***	**Reference group *0*–*2 months n = 104***	**DG versus RG *0*–*2 months (p *value)**	**Diabetes group *2*–*6 months n = 108***	**Reference group *2*–*6 months n = 99***	**DG versus RG *2*–*6 months p *value)**
Breastfeeding rate *n (%)	88 (80.7)	95 (91.3)	0.045^2^	67 (61.5)	79 (76.7)	0.023^2^
Exclusive	72 (80.0)	82 (86.3)	0.325^2^	28 (44.4)	30 (40.5)	0.729^2^
**Breastfeeding complications***n* (%)						
Insufficient milk supply	35 (36.1)	28 (29.5)	0.359^2^	31 (35.2)	31 (28.6)	0.553^1^
Oversupply of breast milk	36 (37.1)	33 (34.7)	0.847^1^	20 (22.7)	17 (16.2)	0.651^1^
Blocked ducts						
Once	10 (10.3)	12 (12.6)	0.656^2^	9 (10.2)	7 (6.7)	0.636^1^
Twice or more	4 (4.1)	11 (11.6)	0.063^2^	4 (4.5)	5 (4.8)	0.553^1^
Cracked nipples	29 (29.9)	52 (54.7)	0.001^2^	10 (11.4)	6 (5.7)	0.494^1^
Painful breastfeeding	34 (35.4)	54 (56.8)	0.009^1^	8 (9.1)	8 (7.6)	0.543^1^
**Breastfeeding experience***n* (%)						
Positive	97 (94.2)	98 (95.1)	1.00^1^	90 (92.8)	89 (90.8)	0.542^1^
**Importance of breastfeeding***n* (%)	*n = 101*	*n = 104*	0.204^1^	*n = 105*	*n = 99*	0.724^1^
Very important /important	77 (76.2)	78 (74.3)		83 (79.8)	74 (74.7)	
Quite important	21 (20.8)	19 (18.1)		21 (20.2)	20 (20.2)	
Not important/ not relevant	3 (3.0)	8 (7.6)		0 (0.0)	5 (5.1)	

^1^ Pearson’s Chi-square ^2^ Fisher’s Exact test * The numbers report on occurrence of breastfeeding at 2 and 6 months interviews. Data reported elsewhere [11].

### Breastfeeding attitudes and complication rates

Of the women with diabetes, 84.1% (n = 90/108) had ‘very high’ or ‘high’ confidence in their breastfeeding capacity before childbirth compared to 92.3% (n = 96/104) women in the reference group; this difference was not significant (p = 0.19). Moreover, there were no differences in attitude regarding the importance of breastfeeding at 2 months or 6 months; a few (3 to 7%) assessed it as ‘unimportant’ (Table 2). Women in the reference group were more likely to have experienced cracked nipples and consequently painful breastfeeding initially (Table 2). Use of pacifiers was more frequent in the diabetes group than in the reference group at 2 months (85.2 vs. 71.2%, p = 0.02) but not at 6 months (77.6 vs. 73.1%, p = 0.51).

### Breastfeeding impact on daily life

The overall experience of breastfeeding was positive for almost all women in both groups (Table 2). Breastfeeding was assessed as influencing daily life ‘very much’ or ‘quite a lot’ during 0 to 2 months by 76.9% of mothers in the diabetes group (n = 83/108) and by 82% of mothers in the reference group (n = 84/104) (p *=* 0.62). At 6 months its influence had dropped; the same assessment being given by 63.1% of mothers with diabetes (n = 65/103) and 58.2% (n = 57/98) of the mothers in the reference group (p = 0.56). This influence significantly dropped over time in both groups (diabetes group: p < 0.01 and reference group: p < 0.01).

Detailed descriptions and a comparison of the impact of breastfeeding on daily structure for mothers in diabetes and reference groups are presented in Table 1. Within the diabetes group, the impact of breastfeeding on the priority of other duties (p < 0.01) and feelings of time pressure decreased between 2 and 6 months (p *=* 0.02). Negative effects on patterns of sleep also decreased during this time (p *=* 0.01). Within the reference group, the only difference between 2 and 6 months after childbirth was being less sensitive to unexpected disruptions in daily routines (p *=* 0.02). Mothers with diabetes remained more affected by disruptions in daily life 2 to 6 months postpartum. At 2 months, mothers in the reference group expressed a greater need to organize their time than mothers in the diabetes group (p = 0.03). Women with diabetes felt more worried about their health during the entire period (p < 0.01 and p = 0.01 respectively) (Table 1).

## Discussion

A high proportion of the women in both groups had felt confident during pregnancy in their capacity to breastfeed. Perhaps this explains, why the mothers with diabetes despite the daily diabetes related pressures, did breastfeed to such a high extent at both 2 and 6 months after childbirth. A high degree of self-efficacy has been found elsewhere to positively influence women’s decision to breastfeed [9]. Another encouraging result in our study is that the overall experience of breastfeeding was positive for almost all women in both groups, and especially for the mothers with diabetes. We have previously reported that these women experience a demanding time during the first few months with their newborn [15]. It would be reasonable to believe that other aggravating circumstances, such as the high frequency of caesarean sections, maternal complications etc., among women with diabetes can have an impact on health outcomes postpartum. However, these variables were not shown to explain the shorter duration of breastfeeding but are likely to explain why initiation and establishment were more delayed in the diabetic group [11].

At both 2 and 6 months after childbirth, mothers with diabetes reported less need to organize their time than before compared to the reference mothers. This may indicate that the mothers with diabetes already had distinct daily structures in place. While everyday life does not have to be highly organized and structured for most people, it often is for mothers with diabetes. As has been described previously [15,22,23,25], for them organizing and predicting everyday life means balancing activities with managing blood glucose levels. This daily challenge might be, at the same time, the explanation for the somewhat inconsistent result that although the mothers with diabetes reported less need to organize their time, they remained more affected by disruptions in daily life at 6 months postpartum than the reference group mothers. Being accustomed to structure as mothers managing their diabetes may have made them more sensitive to changes and disruptions even before they entered motherhood and began breastfeeding. This suggested explanation has to be scientifically proven in future studies.

The study also focused on women’s concerns about their own health, and we found that the mothers with diabetes reported being more worried about their health during the entire study period compared to the reference group. The reason for this was not studied. One explanation might be that their need for insulin, which had increased 2 to 3 times during pregnancy, suddenly decreased afterwards and was frequently less than it was before pregnancy [11]. Hypoglycemic episodes in relation to breastfeeding have also been reported to be more frequent [22], sometimes even dramatic episodes were experienced [15]. Another explanation might be that the increased daily interruptions related to care for the newborn or increased sensitivity to disruptions affected the mothers’ level of concern. This suggested explanation also needs to be further explored.

Another study of a general population of women in Sweden (aged 38 and 50) showed that perceived high frequency of interruptions was related to poor subjective health among the younger women, and to low satisfaction with life as a whole in both groups. Women with children living at home and/or who lived with a partner experienced disturbing interruptions more frequently than those without children living at home or those who lived alone [26]. Because frequent interruptions and changes in daily activities may constitute a risk for a reduction in subjective health, it might be beneficial to advise women with diabetes to minimize the risk for interruptions and changes in routines after childbirth. Our previous findings showed a negative effect on psychological well-being the more breastfeeding affected diabetes management [22]. Since one of the most common sources of interruptions and daily minor hassles in women’s daily life are family members [24], it might be important to inform those living with new mothers with diabetes about their increased sensitivity to interruptions in daily life and an extended need for support to reduce such daily minor hassles to reduce risk of stress and ill health. Previous studies have shown that peer supporters are more likely than health care professionals to provide an authentic presence related to sharing experiences [27]. Mothers with diabetes often lack access to supportive persons with common experiences of diabetes and childbearing [16,28]. Sharing experiences about constructive ways of balancing everyday life and of managing unpredictable blood glucose levels while dealing with daily activities during breastfeeding and childcare might reduce their extraordinary challenges [15].

### Limitations

There are several limitations to be aware of in this study. The relatively small sample size might have implications for the power of the study as statistical power calculations were calculated on variables not central in this study. Another limitation is that due to logistical obstacles we have no information on the drop-out rate in the reference group.

The use of a questionnaire which has not been psychometrically evaluated is a further limitation. We did not find any psychometrically evaluated instrument covering the broad aspects of interest in this study. Unfortunately, the Breastfeeding Self-Efficacy Scale [29] had not yet been translated and validated to a Swedish population. We did, however, test our questionnaire for face validity in both groups of women. Similar statements about daily structure and interruptions have been used in other studies with women [26,30] which may thereby increase the applicability of these statements in the questionnaire. A factor that supports the use of a specifically designed questionnaire is the need to use contextual questions in specific and unique situations. Detailed validation might be needed in some areas. When exploring new areas, a creative and pragmatic approach has to be used, and there are many examples of useful data being derived from simple tools in different studies [31]. Nevertheless, these preliminary findings require replication and further investigation in order to draw conclusions.

The strength of our study is the use of matched controls with same parity and childbirth in the same gestational week, as it allows comparison between two groups with a crucial difference such as living with or without a demanding chronic illness – diabetes, for example.

## Conclusion

The results indicate that mothers with diabetes might be more sensitive to changes and disruptions because of their increased need for daily structure related to managing diabetes particularly in relation to breastfeeding. Based on this and findings from previous studies, one can conclude that they are likely to need additional professional and peer support after childbirth to balance their everyday life to reduce the risk of stress and ill health. Both health professionals and relatives need to be informed of the unique prerequisites of entering motherhood when having diabetes.

## Competing interests

The authors declare that they have no competing interests.

## Authors’ contribution

CS-L collected and analysed data and wrote the manuscript. L-KE wrote the manuscript. MB analysed data and wrote the manuscript. All authors read and approved the final manuscript.
